# Graphomotor memory in Exner’s area enhances word learning in the blind

**DOI:** 10.1038/s42003-021-01971-z

**Published:** 2021-04-06

**Authors:** Tomomi Mizuochi-Endo, Kazuyuki Itou, Michiru Makuuchi, Baku Kato, Kazuhisa Ikeda, Kimihiro Nakamura

**Affiliations:** National Rehabilitation Center for Persons with Disabilities, Tokorozawa, Japan

**Keywords:** Language, Reading

## Abstract

Handwriting is thought to impede vocabulary learning in sighted adults because the motor execution of writing interferes with efficient audiovisual processing during encoding. However, the motor memory of writing may facilitate adult word learning when visual sensory inputs are severely restricted. Using functional MRI, we show that late-blind participants, but not sighted participants, learned novel words by recruiting the left dorsal premotor cortex known as Exner’s writing area and its functional coupling with the left hippocampus. During later recall, the phonological and semantic contents of these words are represented in the activation patterns of the left hippocampus as well as in those of left frontotemporal language areas. These findings suggest that motor codes of handwriting help blind participants maintain word-form representations during learning and retrieval. We propose that such reliance on the motor system reflects a broad architecture of the cerebral language network which encompasses the limb motor system as a hardwired component.

## Abstract (web summary)

Mizuochi-Endo et al. conduct a fMRI study, which reveals that in blind participants, unlike sighted participants, learning new words is associated with increased activity in Exner’s area—a part of the brain known to play a crucial role in handwriting motor memory. This demonstrates the importance of writing motor memory in vocabulary learning in the blind.

## Introduction

Handwriting is a complex psychomotor skill that engages a broad set of neurocognitive systems, including motor, semantic, and phonological memory^1,2^. While the use of paper-and-pencil seems to be increasingly less common today, handwriting is known to play an important role in literacy development. Namely, a body of behavioral studies with young children indicates that handwriting practice facilitates visual letter/word recognition across different languages^3–8^. Such facilitatory effects are thought to arise because learning while writing can effectively activate neurocognitive systems required for reading, i.e., cognitive control, attention, visuospatial analysis, and phonological processing^8^. These observations suggest that the motor memory of handwriting promotes visual word processing and seem to concur with the notion that multisensory training can produce more efficient learning in skill acquisition than unisensory training^9^. Handwriting may also exert facilitatory effects on vocabulary learning, since a recent cross-linguistic study by Cao et al.^10^ has shown that handwriting-based learning outperforms speech-based learning when Chinese and English adults learned unknown Spanish words.

In fact, however, effects of writing seem much less consistent in adult word learning than those observed in young children. In particular, a series of behavioral studies by Barcroft^11–13^ show that vocabulary learning measures are lower when adult participants wrote target words than when they did nothing while learning. These negative effects of writing are interpreted as suggesting that handwriting forces motor output without access to meaning and detracts from word learning by exhausting processing resources needed to encode novel lexical forms. This seems plausible because learning while writing may require difficult multi-tasking, which can exceed well-known capacity limits in information processing during central executive control^14^. Accordingly, the existing behavioral data suggest that handwriting exerts beneficial effects during word learning in children but not necessarily in adults. If this is the case, however, why does the act of writing help word learning less effectively in adults than in children?

One possible account is that handwriting acts as a trade-off against audiovisual learning in adults but not in children. That is, adult learners are likely to rely on visual and phonological memory systems to create novel associations between word form and meaning, which can be simply impeded when attentional resources are partially assigned to the motor execution of writing. It is possible that literate adults are predisposed to such visual and phonological resources under the strong influence of literacy^15^, which is known to yield tight reciprocal connections between visual and auditory systems^16^. Indeed, the level of reading skill is shown to influence lexical learning rate in adult word learning^17,18^. On the contrary, preschool learners may rely on visual and phonological systems to a lesser degree and thus exploit the motor memory of writing more effectively during word learning. This is possible because novel word learning heavily relies on the working memory system^19^, whose visual and auditory storage systems, as well as their executive control, are known to develop gradually from early childhood to adolescence^20,21^. If this is the case, motor codes of writing would play a greater role in word learning for those with limited access to written text, such as dyslexic and blind people. In fact, neuroimaging data with dyslexic children suggest that reading difficulties are partially compensated by relying on Exner’s area^22^ or a left dorsal premotor region (PMd) known to represent the motor memory for handwriting^23^. However, such reliance on the motor system may reflect not only specific compensatory change in the dyslexic brain but also a more universal and intrinsic architecture of the human language network, because the same left PMd is also shown to mediate novel word learning in normal adults via motor memory^24^ and play a role in sign language processing^25,26^. It is therefore possible that, even in adulthood, motor codes of writing exert beneficial effects on word learning when visual or auditory sensory inputs are restricted. In particular, while the crossmodal interaction between the visual and phonological systems is thought to play a primary role in adult word learning^27,28^, such learning mechanism enhanced by literacy should be compromised by the long-term deprivation of visual inputs in the late-blind, which can attenuate visual memory retrieval^29,30^ but may allow more cognitive resources for exploiting motor codes during word learning. We thus hypothesized that Exner’s area involved in the motor memory for handwriting should play a greater role in word learning for those blind people than for sighted people.

**Fig. 1:**
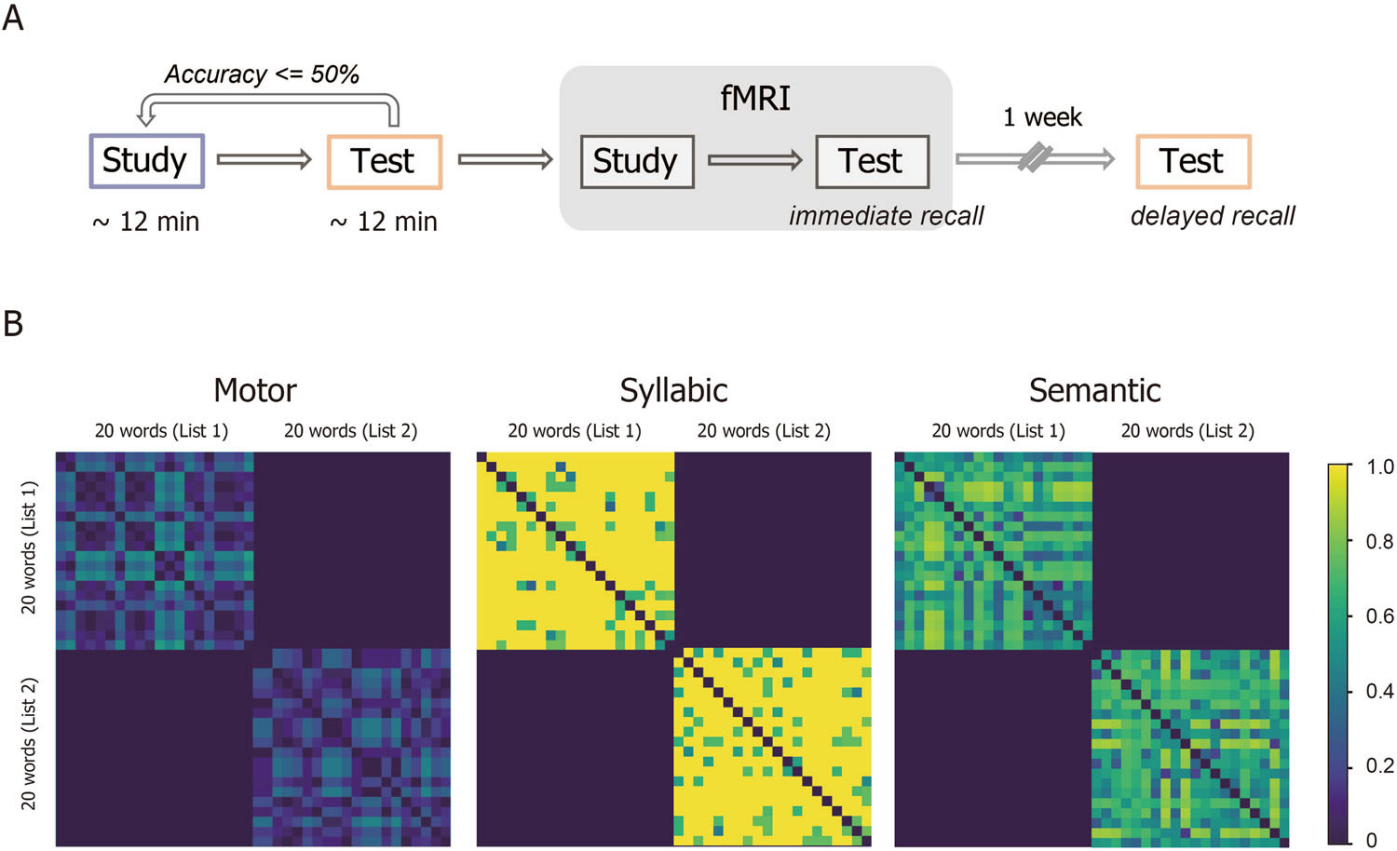
Experimental paradigm. **A** The behavioral paradigm consisted of several study–test cycles for foreign language (FL) word learning. Participants first heard and learned a list of 20 word pairs in a study period and then recalled the meanings of FL words in a test period. In the writing condition, participants studied each FL word while spelling it out with the right index finger, while in the no-writing condition, they studied the target word while keeping the right hand relaxed. The two conditions were performed in two separate sessions spaced at least 3 weeks from each other. During the test period immediately after the study period, participants were presented with FL words and asked to recall their meanings. This cycle of study and test was repeated outside the MRI scanner until participants achieved >50% accuracy in the test period. Participants then lay in the MRI scanner and received one additional cycle of study and test during fMRI scanning. Spoken responses were recorded and analyzed as “immediate recall.” One week after fMRI scanning, participants received the same recall test for each condition (“delayed recall”). **B** Three theoretical representational dissimilarity matrices (RDMs) used for multi-voxel pattern analysis. The motor RDM was created to characterize motor-level dissimilarity in writing movements between FL words. Pairwise comparisons of all possible word pairs yielded the motor RDM as a 40 × 40 symmetrical matrix of which the off-diagonal values represent the dissimilarity for each pair of words at the motor level. For the syllabic RDM, syllabic dissimilarity between a pair of words was calculated as the proportion of shared syllables between the two words, yielding a 40 × 40 matrix whose off-diagonal values represent pairwise dissimilarities between words at the syllable level. Likewise, the 40 × 40 semantic RDM was constructed by computing semantic distance between words according to the taxonomy provided by the WordNet (see “Methods”).

In the present study, we used event-related functional magnetic resonance imaging (fMRI) to examine behavioral and neural effects of handwriting on word learning in late-blind and sighted participants. Each participant received a set of fMRI experiments, which consisted of a study period and a test period (Fig. 1A). During the study period, participants heard unknown foreign language (FL) words and learned them in two different conditions, i.e., they either wrote down target FL words (“writing” condition) or kept their arm and hand muscles relaxed (“no-writing” condition) while learning. Participants then received a test period in which they recalled the meaning of learned FL words. In general linear model (GLM) analyses, we searched for brain regions showing differential activations associated with writing between blind and sighted participants and then examined their functional connectivity during learning and retrieval. We then performed representational similarity analysis (RSA, see Fig. 1B)^31^ to identify the representational content of those brain regions that emerged during the test period.

## Results

### Behavioral results

**Fig. 2:**
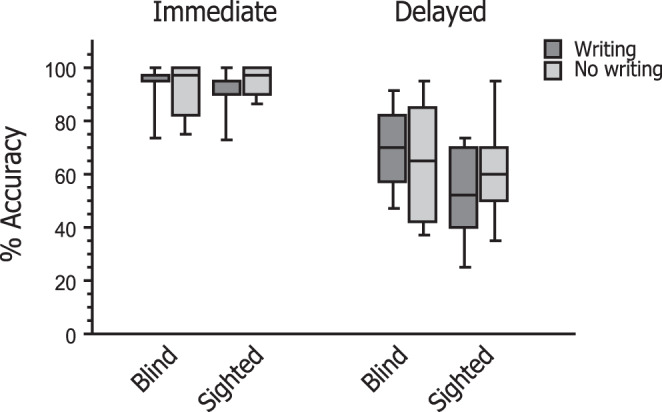
Behavioral results during the test period. During immediate recall in the MRI scanner, blind and sighted participants identified FL word meanings with high accuracy (>90%) irrespective of the learning conditions. On delayed recall, however, blind participants tended to score higher in the writing than in the no-writing condition, whereas sighted participants showed an opposite trend between the writing and no-writing conditions. The differential impact of writing was confirmed as a robust interaction between the effects of condition and group (see “Results”). In this and all subsequent figures, box plot elements are defined as follows: center line, median; box limits, upper and lower quartiles; whiskers, 1.5× interquartile range.

Box plots of accuracy data for the immediate and delayed recall tests are illustrated in Fig. 2. During immediate recall, blind participants identified FL word meanings with accuracy level (SD) of 92.50% (9.65) in the writing condition and 92.08% (10.33) in the no-writing condition. Sighted participants performed the same task with accuracy level of 90.27% (11.04) in the writing condition and 95.28% (6.06) in the no-writing condition. On delayed recall, blind participants tended to score higher in the writing than in the no-writing condition (69.58 vs. 64.58%), whereas sighted participants showed an opposite trend between the writing and no-writing conditions (52.22 vs. 62.22%).

To validate these observations, we assessed the accuracy data using nonparametric aligned rank transformation analysis of variance (ANOVA)^32,33^, which included learning condition (writing vs. no-writing), test timing (immediate vs. delayed) as within-participant factor, and group (blind vs. sighted) as a between-participant factor. The effect of test timing was highly significant (*F*[1,28] = 91.42, *η*^2^ = 0.77, *p* = 2.57 × 10^–10^), indicating that participants scored higher on immediate recall than on delayed recall. The effect of condition was significant (*F*[1,28] = 6.33, *η*^2^ = 0.18, *p* = 0.018), suggesting that participants overall scored higher in the writing relative to the no-writing conditions. The effect of group was also significant (*F*[1,28] = 6.04, *η*^2^ = 0.184, *p* = 0.020), suggesting that blind participants overall scored higher than sighted participants. Notably, there was a highly significant interaction between the effects of condition and group (*F*[1,28] = 13.70, *η*^2^ = 0.33, *p* = 9.31 × 10^–4^), validating the differential impact of writing as described above. In addition, the effect of timing interacted with that of group (*F*[1,28] = 9.28, *η*^2^ = 0.25, *p* = 0.005) but not with that of condition (*F*[1,28] = 3.80, *η*^2^ = 0.12, *p* = 0.061). There was a significant three-way interaction between the effects of timing, condition, and group (*F*[1,28] = 9.70, *η*^2^ = 0.26, *p* = 0.004).

In subsequent analyses, we assessed the relative contribution of each group to the observed condition × group interaction by testing the effects of condition and timing separately for each group. In the blind group, the effect of timing was highly significant, with the accuracy level being higher for immediate than for delayed recall (*F*[1,11] = 38.47, *η*^2^ = 0.78, *p* = 6.69 × 10^–5^). The effect of condition was borderline significant (*F*[1,11] = 4.72, *η*^2^ = 0.30, *p* = 0.053), whereas the interaction between timing and condition did not reach significance (*F*[1,11] = 3.70, *η*^2^ = 0.25, *p* = 0.081). These findings thus suggest that blind participants overall scored higher in the writing than in the no-writing condition across the immediate and delayed recall tests. As for the sighted group, the effects of timing and condition were both significant (*F*[1,17] = 67.75, *η*^2^ = 0.80, *p* = 2.47 × 10^–7^ and *F*[1,17] = 8.63, *η*^2^ = 0.58, *p* = 0.009, respectively). Since these effects interacted with each other (*F*[1,17] = 4.48, *η*^2^ = 0.21, *p* = 0.049), we further examined the effects of condition separately for each test timing. We found that this effect was significant for immediate recall (*F*[1,17] = 6.86, *η*^2^ = 0.29, *p* = 0.018) and not for delayed recall (*F*[1,17] = 1.22, *η*^2^ = 0.07, *p* = 0.285). This finding can be attributed to the much larger variability in accuracy during delayed recall as compared to immediate recall in the sighted group (see Fig. 2). Accordingly, these post hoc analyses show that the critical condition × group interaction during immediate recall was contributed by both groups. Taken together, the present behavioral results overall suggest that handwriting movements exert beneficial effects on word learning in blind participants but not in sighted participants.

### GLM results

**Fig. 3:**
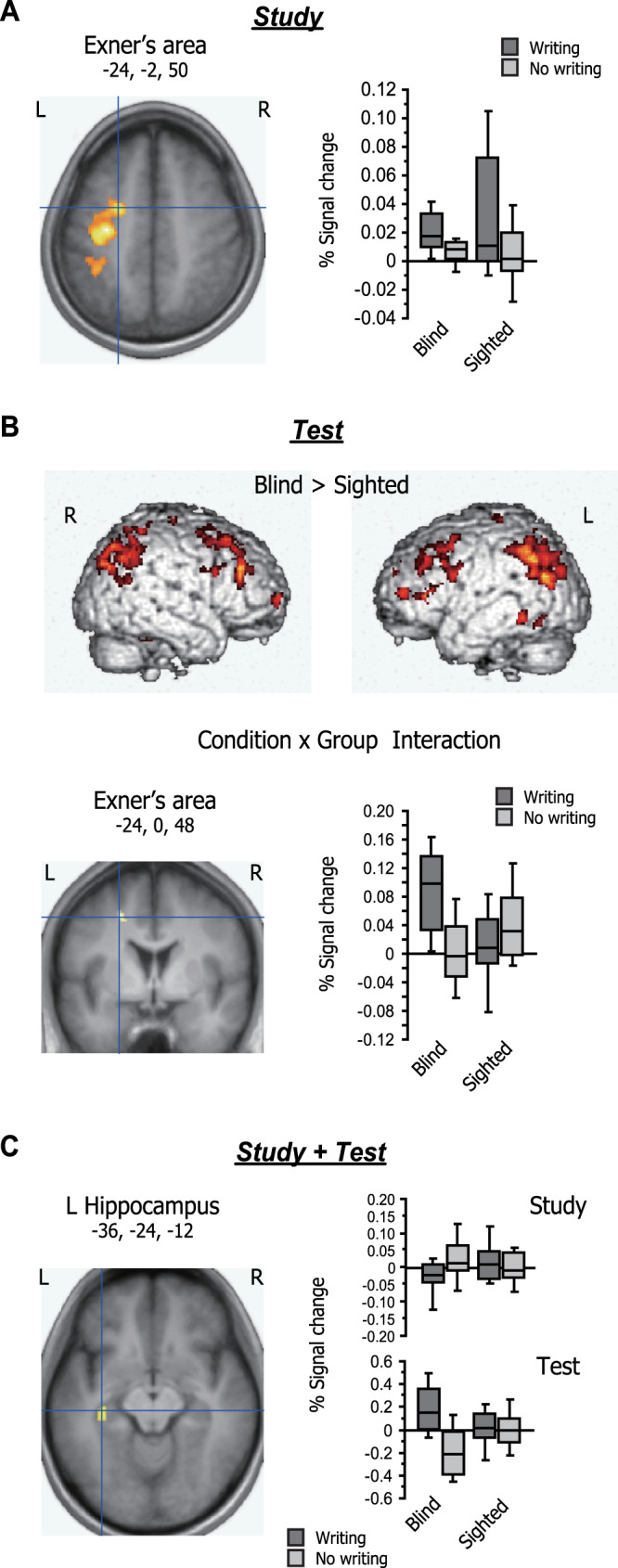
GLM results. **A** Neural effects of learning condition and group during the study period. The main effect of condition calculated in the writing vs. no-writing contrast was observed in the right cerebellum (30, -50, −26, *Z* = 4.88) and the left sensorimotor cortex (−32, −18, 50, *Z* = 6.50), which extended to the known coordinates of Exner’s area (left). No brain region showed the effect of group, either in the whole-brain analysis or in the ROI analyses (*p* > 0.1 for all). Significant interaction between condition and group was found in Exner’s area ROI (−20, −4, 52, *p* = 0.013, right). **B** Neural effects of condition × group interaction during the test period. The main effect of group was observed in bilateral frontal and parietotemporal regions showing greater activation in blind relative to sighted participants (top). In ROI analysis, Exner’s area showed a greater effect of learning condition for blind than for sighted participants, creating significant condition × group interaction (−24, 0, 48, *p* = 0.003, bottom). Note that this interaction was reversed in direction from the one observed during the study period. **C** Psychophysiological interaction with Exner’s area. The magnitude of connection strength is represented as percent signal change relative to the baseline. A middle part of the left hippocampus (−36, −24, −12, *Z* = 3.78) was identified as showing increased functional connectivity with Exner’s area across the study and test periods.

We first examined the effects of condition and group and their interaction during the study period. Both the writing and no-writing conditions strongly activated the bilateral temporal lobe relative to the baseline. The main effect of condition, i.e., activation difference associated with writing, was significant in the right cerebellum (30, −50, −26, *Z* = 4.88, 201 voxels) and the left sensorimotor cortex (−32, −18, 50, *Z* = 6.50, 2208 voxels), which extended to the previously reported coordinates of Exner’s area^23^ (Fig. 3A). This finding is in good accordance with a recent meta-analysis, which identified these structures as specific neural systems for handwriting^34^. In the region-of-interest (ROI) analyses, the effect of writing was again highly significant in Exner’s area (*Z* = 5.10, *p* < 0.00001) but non-significant in the left fusiform visual word-form area (VWFA^35^, *p* > 0.1). In contrast, no brain region showed the effect of group, either in the whole-brain analysis or in the ROI analyses (*p* > 0.1 for all). Significant interaction between condition and group was observed only in the left frontoparietal junction area (−36, −22, 56, *Z* = 4.74, 209 voxels), which showed a greater effect of writing for sighted than for blind participants. As for ROI analyses, the same effect was significant in Exner’s area (−20, −4, 52, *p* = .013, see Fig. 3A) but non-significant in VWFA (*p* > 0.1). The effects of condition and group and their interaction were all non-significant in the left and right hippocampal ROIs (*p* > 0.1 for all).

We then assessed the same effects and interactions during the test period. The effect of group was broadly distributed in bilateral frontal and parietotemporal regions, which showed greater activation in blind relative to sighted participants (Fig. 3B). In ROI analyses, this effect of group was also significant in both Exner’s area (−20, −2, 48, *p* = 0.009) and VWFA (−50, −52, −10, *p* = 0.001). However, the effect of learning condition was non-significant throughout the entire search volume, both in whole-brain analysis and in ROI analyses (*p* > 0.1 for all). As for the condition × group interaction, although no brain region survived the conservative whole-brain analysis, we observed significant interaction in Exner’s area ROI (−24, 0, 48, *p* = 0.003, Fig. 3B), which showed a greater effect of learning condition for blind than for sighted participants. Note that this condition × group interaction was reversed in direction relative to the one observed during the study period. There was a non-significant trend of interaction in the VWFA (−46, −54, −8, *p* = 0.072).

Based on the observed condition × group interaction in Exner’s area during the study and test periods, we ran psychophysiological interaction (PPI) analyses to search for brain regions showing the parallel change in functional connectivity with this area across the two periods (thresholded at voxel *p* < 0.001, cluster extent >10 voxels). This analysis identified a middle part of the left hippocampus (−36, −24, −12, *Z* = 3.78, 18 voxels, Fig. 3C). A weaker trend was also observed in the right homologous area (32, −28, −12, *Z* = 3.19, 4 voxels). ROI analyses further confirmed significant condition × group interaction in the middle segment of the left hippocampus (*p* = 0.028) but not in other segments of the left and right hippocampus (*p* > 0.1 for all). In addition, we observed the similar change in functional connectivity between Exner’s area and VWFA (−40, −60, −16, *p* = 0.052).

### RSA results

**Fig. 4:**
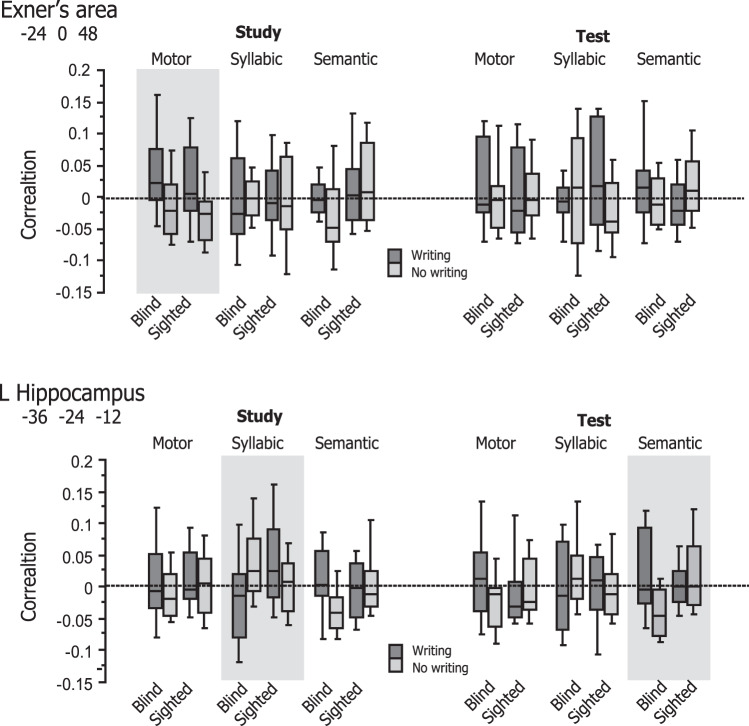
Correlations with the theoretical RDMs in Exner’s area and left hippocampus. During the study period, multi-voxel activation patterns in Exner’s area showed a significant effect of learning condition for the motor RDM (*p* = 0.013, shaded in gray). The main effects and their interaction were non-significant for the three RDMs during the test period (*p* > 0.1 for all). On the other hand, the left hippocampus showed significant crossover interaction with the syllabic RDM (*p* = 0.023), suggesting that handwriting yielded a differential impact on activation patterns between the blind and sighted groups. During the test period, the same segment of the left hippocampus showed significant condition × group interaction for the semantic RDM (*p* = 0.026), again suggesting that semantic information was more weakly represented for the blind relative to the sighted participants.

We then performed RSA to assess the representational content of multi-voxel activation patterns for Exner’s area and the left hippocampus identified in GLM analyses (Fig. 4). We first selected those voxels in Exner’s area showing condition × group interaction (thresholded at *p* < 0.005, 34 voxels) and assessed whether their activation patterns were sensitive to the motor, syllabic, and semantic representational dissimilarity matrices (RDMs) during the study period. For the motor RDM, we obtained a significant effect of condition (*p* = 0.013, whereas the effect of group and the condition × group interaction were both non-significant (*p* > 0.1 for both), suggesting that activation patterns in Exner’s area correlated with the motor RDM in the writing condition more strongly than in the no-writing condition across the two participant groups. On the other hand, the two main effects and their interaction were non-significant for the syllabic RDM (*F* < 1 for all). As for the semantic RDM, there was a non-significant trend of group (*p* = 0.078), with the correlation strength being greater for sighted than for blind participants. The effect of learning condition and its interaction with group were non-significant (*p* > 0.3). Taken together, these findings suggest that Exner’s area was sensitive to the motor RDM but not to the syllabic and semantic RDMs during the study period. By contrast, the two main effects and their interaction were all non-significant for the three RDMs during the test period (*p* > 0.1 for all).

We next looked at the left hippocampus that showed significant change in functional connectivity with Exner’s area (thresholded at *p* < 0.005, 53 voxels). For the study period, the two main effects and their interaction were non-significant for the motor RDM (*F* < 1 for all). The effects of condition and group were both non-significant for the syllabic RDM (*F* < 1 for both), but their interaction was significant (*p* = 0.023). This crossover interaction suggests that the effect of learning condition yielded a differential impact on activation patterns between the blind and sighted groups (see Fig. 4). For the semantic RDM, the two main effects were also non-significant (*p* > 0.2 for both), whereas their interaction showed a weak trend (*p* = 0.084), suggesting that semantic information was more weakly represented for the blind relative to the sighted participants. As for the test period, the semantic RDM showed no significant effects of group and condition (*p* = 0.092 and *p* > 0.5, respectively), whereas these effects interacted with each other (*p* = 0.026), again suggesting that semantic information was more weakly represented for the blind relative to the sighted participants. The two main effects were also non-significant for the motor RDM (*p* > 0.5 for both) but their interaction was marginally significant (*p* = 0.071). The main effects and their interaction were all non-significant for the syllabic RDM (*p* > 0.3 for all).

**Fig. 5:**
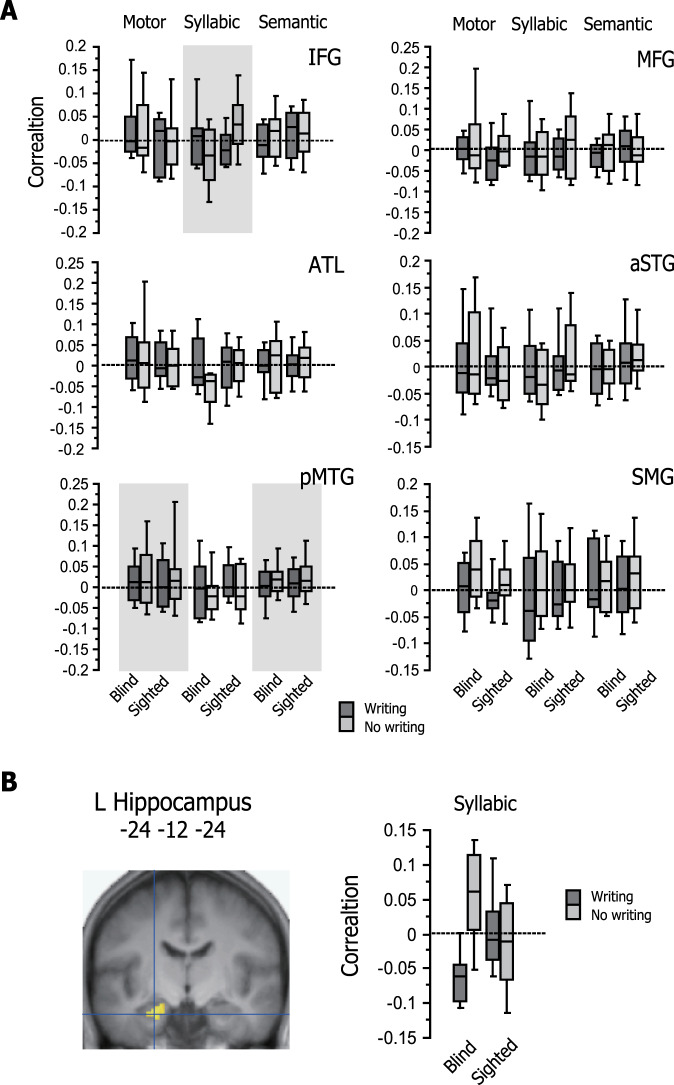
Correlations with the theoretical RDMs during the test period. **A** Left hemisphere ROIs. The left pMTG overall showed significant correlation with the motor and semantic RDMs (*p* = 0.050 for both, shaded in gray). On the other hand, the left IFG showed significant condition × group interaction (*p* = 0.030, shaded in gray) for the syllabic RDM. In the left SMG, there was a weak trend of writing with the syllabic RDM (*p* = 0.115). No other ROI showed significant main effects or their interactions with any of the three RDMs (*p* > 0.2 for all). For each ROI, box plots of *Z*-transformed Spearman correlation were calculated at the voxels showing the maximum effect for each RDM. **B** Searchlight RSA. Significant condition × group interaction for the syllabic RDM was found in the left IFG (−52, 26, 10, *Z* = 4.11) and the left hippocampus that extended from the anterior to middle segments (−24, −14, −24, *Z* = 3.99 and −28, −24, −16, *Z* = 3.67, respectively).

To explore between-group differences in neural outcomes after learning, we additionally selected six regions of the left hemisphere language network and looked at their informational content during the test period (Fig. 5A). For each ROI, we first asked whether overall correlation strength with neural RDMs differed from zero across participants for each of the three theoretical RDM. We observed that the left posterior middle temporal gyrus (pMTG) overall showed significant correlation with the motor and semantic RDMs (*p* = 0.050 for both). None of the other ROIs correlated with any of the three RDMs (*p* > 0.15 for all). Next, we examined the effects of writing and group on the correlation strength for each RDM. For the syllabic RDM, we observed that the left inferior frontal gyrus (IFG) showed significant condition × group interaction (*p* = 0.030). In the left supramarginal gyrus (SMG), there was a weak trend of writing with the syllabic RDM (*p* = 0.115). No other ROI showed significant main effects or their interactions with any of the three RDMs (*p* > 0.2 for all).

Lastly, we performed searchlight RSA to search for other neural systems showing sensitivity to the three theoretical RDMs during the test period. This analysis identified several regions showing condition × group interaction for the syllabic RDM, including the left IFG (−52, 26, 10, *Z* = 4.11, 74 voxels) and an anterior segment of the left hippocampus (−24, −14, −24, *Z* = 3.99, 74 voxels, Fig. 5B). There was marginally significant interaction in the left ventral premotor cortex (−42, 2, 36, *Z* = 4.09, 61 voxels (cluster p = 0.057)). This interaction was significant in Exner’s area ROI (−26, −6, 50, *p* = 0.007) but not in the VWFA ROI (*p* > 0.1). Other regions showing significant condition × group interaction included the right ventral premotor area (60, 4, 10, *Z* = 4.93), right middle frontal gyrus (MFG; 22, 28, 48, *Z* = 4.44), left cerebellum (−40 −76 −50, *Z* = 4.67), and left medial frontal area (−2, 50, −14, *Z* = 4.53). No other regions showed significant effects or interactions with any of the three RDMs (*p* > 0.1 for all).

## Discussion

Vocabulary is a building block of the language faculty that links seemingly arbitrary linguistic signs with semantic memory. By early adulthood, average language users are estimated to know ≥50,000 words, a vocabulary size >100 times greater than known capacity limits in non-human primates^36^. A well-known cognitive model for vocabulary learning is fast mapping in young children^37^, which may be also functioning in adults and likely relies on the left perisylvian language cortex^38,39^. Yet vocabulary acquisition may capitalize on other neural systems when audiovisual inputs are severely restricted because of peripheral sensory disorders. In the present study, we predicted that the graphic motor memory for writing in Exner’s area would benefit word learning more greatly in blind people than in sighted people.

Indeed, our behavioral results from the immediate recall test have shown that blind participants, but not sighted participants, tended to score higher in the writing relative to the no-writing condition. On the one hand, this finding is consistent with the behavioral studies with sighted adults showing that handwriting detracts from word learning by exhausting processing resources for encoding novel lexico-semantic association^11–13^. The motor execution of writing may interfere with word learning only during early encoding, since we observed that this negative effect of writing was no longer distinguishable during delayed recall.

On the other hand, the positive effect of handwriting observed in blind participants fits with the previous studies with preschoolers and dyslexic children showing that motor codes of writing can facilitate word learning. As predicted before, handwriting can rather impede word learning in literate adults, because spoken or written words automatically trigger crossmodal activation of the visual and phonological memory systems^40^, which in turn should occupy the cognitive or attentional resources available for encoding novel words. In contrast, motor codes of words can facilitate word learning in blind adults, preschoolers, and dyslexic children, likely because spoken word inputs can produce only unimodal activation of phonological systems, leaving the cognitive resources available to exploit motor codes of words. This interpretation also seems consistent with the recent behavioral data from deaf children, which showed a greater beneficial effect of handwriting in deaf children than in typically developing children^41^. Thus, the present results may reconcile the seemingly conflicting effects of handwriting on word learning among sighted adults, preschool children, and people with audiovisual disabilities.

At the neural level, our fMRI results for the study period revealed that the left frontoparietal region, including Exner’s area, showed not only significant effect of learning condition (i.e., greater activation in the writing relative to the no-writing condition) but also significant condition × group interaction (i.e., greater effect of writing for sighted than for blind participants). This condition × group interaction during study can be taken as reflecting that handwriting is a less preferred sensory pathway for word learning for sighted participants, which lead to more effortful recruitment of Exner’s area as compared to blind participants. During the test period, however, the same Exner’s area showed significant condition × group interaction in the opposite direction, i.e., a greater effect of writing for blind than for sighted participants.

Accordingly, a key finding from the present GLM analyses is that Exner’s area showed the condition × group interaction not only during the study period but also during the test period. Given the known role of Exner’s area in writing^23,42,43^, this finding indicates that blind participants rely on the motor memory for handwriting differently from sighted participants during word learning and retrieval. Importantly, however, such reliance on motor codes is probably functioning not as a mere compensatory neurocognitive mechanism for blind people but as a more intrinsic neural pathway for word learning in the human brain. In fact, the similar motor memory for body movements is likely to be functioning in sighted adults, that is, the left PMd close to the present Exner’s area ROI (−23, −15, 51 in the Montreal Neurological Institute (MNI) space) is shown to mediate word learning via iconic gestures^24^. Moreover, visual and motor systems outside the classical left hemisphere language network are shown to contribute to vocabulary learning in sighted adults^44^.

In PPI analysis, we further found that Exner’s area showed parallel change in functional connectivity with a middle segment of the left hippocampus across the study and test periods (see Fig. 3C). During the test period, in particular, blind participants but not sighted participants showed increased connection strength in the writing relative to the no-writing condition. While the hippocampus is known to receive projections from multiple cortical areas including the sensorimotor cortex^45,46^, its functional connection with the premotor area seems to play a key role in motor learning^47^. The present finding therefore suggests that blind participants, but not sighted participants, capitalized on the neural circuitry for motor learning even during the immediate recall test, which did not require any overt hand movements. Since the middle or intermediate part of the hippocampus has been associated with associative learning and retrieval^48–50^, the present finding is also in good accordance with neurocognitive models of word learning, which encompass the hippocampus and other neocortical areas during word memory trace formation^38,51,52^.

As predicted, the present RSA analyses confirmed that multi-voxel patterns in Exner’s area are sensitive to the motor RDM during the study period. We further observed that activation patterns of the left hippocampus correlated with the syllabic RDM but not with the motor and semantic RDMs, suggesting that this region is sensitive to the phonological information during the study period. Again, this finding concurs with neurocognitive models of vocabulary acquisition whereby the hippocampus plays a pivotal role in the initial phonological encoding of novel words^51,52^. During the test period, on the other hand, the neural RDM of the left hippocampus showed significant correlation with the semantic RDM but not with other RDMs (see Fig. 4). The observed sensitivity to the semantic information is also consistent with the well-known role of the hippocampus in rapid memory consolidation^53^ and likely to reflect the functional requirement during the test period (i.e., immediate recall), where participants needed to access the meanings of newly learned FL words. The present searchlight RSA further revealed that an anterior segment of the left hippocampus was sensitive to the syllabic information during the test period (see Fig. 5B). Coupled with the PPI results described above, these findings from RSA analyses suggest that blind participants, but not sighted participants, encode and retrieve the phonological and semantic content of novel words by modulating the functional connectivity between the left hippocampus and Exner’s area. Moreover, given the role of Exner’s area in graphomotor memory^23^, motor codes of writing may help blind participants maintain form representations of FL words during learning and retrieval. This interpretation seems to concur with another PPI finding that Exner’s area showed parallel change in functional connectivity with the VWFA, which is known to house abstract representations of word forms^35^.

The present RSA analyses for the test period revealed a condition × group crossover interaction in correlation strength between the left IFG and the syllabic RDM (see Fig. 5A). For sighted participants, activation patterns in this area therefore reflected phonological information more strongly in the no-writing condition than in the writing condition, whereas this impact of writing was reversed for blind participants. This differential effect of writing on phonological processing is consistent with the behavioral effects of writing on word learning performance and supports the notion that handwriting detracts from word learning by exhausting processing resources needed to encode novel lexical forms^11–13^. Together with the syllabic information represented in the left anterior hippocampus, this finding corroborates the neurocognitive models of word learning in which the left IFG works in conjunction with the medial temporal region during phonological encoding and retrieval^51,52^.

We also observed that activation patterns in the left pMTG correlated with the semantic and motor RDMs during the test period. Since the effects of condition and group were non-significant for these RDMs, this region exhibited sensitivity to both the motor codes and semantic contents of FL words irrespective of conditions and groups. While the left pMTG has been shown to play a role in mapping lexical word forms onto meaning during visual word recognition^54,55^, the observed sensitivity to the semantic similarity is in line with more recent studies showing that the same region also contributes to novel word learning by mediating form-to-meaning mapping^52,56–58^. In this context, a possible account for the concurrent sensitivity to the motor RDM is that motor stroke patterns in writing can serve as lexical or word-form representations during novel word learning, as argued above. Interestingly, however, the pMTG is also known to include a neuronal cluster involved in the storage and retrieval of action knowledge^59^. The present finding may be therefore related to the fact that our ROI for the pMTG (−56, −36, −2) partially overlaps the “biological motion superior temporal sulcus” (−54, −43, −8), which is shown to work with the PMd to facilitate word learning via motor memory for gestures^44^. On this account, the left pMTG can represent the motor information of stimuli since this part of the lateral temporal cortex encompasses neuronal populations sensitive to biological motion, such as gestures and handwriting. Given that the PMd represents abstract codes of such learned motor acts, it may be further argued that the left pMTG serves for linking word meanings with graphic motor codes in the PMd during word learning.

There are a few limitations of the study that should be considered. First, the relatively small sample size (12 blind and 18 sighted participants) in the present study may limit the reliability and generalizability of the behavioral and neural effects obtained. Second, while we relied on the notion that the long-term deprivation of visual inputs compromises visuospatial memory in the late-blind^29,30^, it should be further explored in future research how far residual visual memory contributes to word processing in those people. Third, our sample might consist of a heterogeneous group of blind participants since two of them had some experience in Braille reading. We nevertheless believe that our main findings are most likely valid because tactile sensory activation associated with Braille reading skills can occupy cognitive resources for word learning only to a limited degree, especially during handwriting. That is, Braille reading in itself is much slower and more effortful than print reading (e.g., even a good Braille reader can read at less than half the speed of a print reader^60,61^). Thus, possible spontaneous activations of tactile memory in Braille users should be much slower and weaker as compared to the automatic visual and phonological activation in sighted adults. Moreover, Braille reading and handwriting rely on heavily overlapping neural systems for hand sensorimotor control. In the writing condition, these systems thus should be massively occupied by the motor execution of writing, which overrides any spontaneous activation of tactile memory in Braille users.

In summary, the present study suggests that motor codes of writing can facilitate novel word learning when visual sensory inputs are restricted in adults. Our fMRI results show that this between-group difference in the impact of writing is mediated by Exner’s area coding for handwriting memory. We also found that blind participants encode and retrieve the phonological and semantic content of novel words by modulating the functional connectivity between the left hippocampus and Exner’s area. Despite the similar level of behavioral accuracy during learning, blind and sighted participants thus relied on different neural mechanisms as reflected in the activation and connectivity in these structures. While vocabulary acquisition is thought to rely on multiple neural systems in the developing and mature human brain^51,52^, these findings are generally in line with the notion that the graphomotor memory in Exner’s area promotes language development and processing in preschoolers^3,62^, dyslexic children^22^, and neurological patients with alexia^63^. Yet the same PMd is also known to play a role in sign language processing^25,26^ and likely to enhance word learning in normal adults when they learn novel word meanings with symbolic gestures^24,44^. Therefore, the left premotor cortex involved in hand movements, although located outside the classical left hemisphere language network, may well partially take over complex cognitive processing like word learning. This tight functional coupling between the hand motor system and the language network seems dormant and unobservable during language processing in healthy people but can be uncovered once a part of the normal language network is compromised. Our results extend the existing knowledge by showing that the same neurocognitive mechanism is effectively functioning during word learning in adulthood even when novel word forms have no inherent association with their meanings. Taken together, these different lines of behavioral and neuroimaging data converge to suggest that such reliance on the motor system is not specific compensatory change in brain dysfunctions but reflects a broader architecture of the cerebral language network than thought previously, which encompasses the limb motor system as its pivotal hardwired component.

## Methods

### Participants

Twelve late-blind participants (two females, mean ± SD age: 33.83 ± 10.26 years) were recruited at the Training Center for Independent Living, National Rehabilitation Center for Persons with Disabilities (NRCD). A separate age- and education-matched group of 18 sighted participants were also recruited (eight females, mean ± SD age: 29.39 ± 9.48 years). All of them were native monolingual speakers of Japanese and strongly right-handed as assessed by the Flinders Handedness Survey^64^ (mean ± SD score: 95.00 ± 6.46 for the blind and 99.17 ± 5.00 for the sighted). Clinical characteristics of the blind participants are summarized in Table 1. All of them had met the criteria for severe visual disabilities (Grade I or II defined by the Japan Ministry of Health, Labor and Welfare, i.e., low visual acuity (<20/200 in the better eye) or severe visual field defect (<10° for each eye)) at least 2 years prior to the experiment. Since total blindness occurs rarely in the late blind, all could perceive light and recognize shape to some extent. Two of them (participants 7 and 9) could read and write with Braille. None of them had known neurological or psychiatric disorders. All gave written informed consent prior to the experiments. The protocol of this study was approved by the institutional ethics committee of the NRCD.

**Table 1:** Demographics of the blind participants.

ID	Age (years)	Sex	Education (years)	Onset	Cause of blindness
1	27	M	16	10	Retinitis pigmentosa
2	22	M	14	20	Hereditary optic neuropathy
3	34	M	12	30	Glaucoma
4	38	F	16	13	Retinitis pigmentosa
5	53	M	16	47	Optic nerve atrophy
6	43	M	16	30	Retinitis pigmentosa
7	24	M	12	14	Congenital glaucoma
8	25	M	12	14	Retinitis pigmentosa
9	40	M	14	15	Retinitis pigmentosa
10	20	M	12	18	Hereditary optic neuropathy
11	32	F	12	7	Retinitis pigmentosa
12	48	M	12	46	Diabetic retinopathy

### Behavioral paradigm

We selected 40 Japanese words each paired with unknown FL words (20 Finish words and 20 Ainu words). Each of the FL words was written with 2–5 characters in kana script (a Japanese syllabary comprising 48 characters and diacritic marks). We then created 2 lists of 20 word-pairs (List 1 and List 2) each of which included 10 Finish words and 10 Ainu words. Mean word length (SD) was 3.15 (0.79) characters for List 1 and 3.05 (0.80) for List 2, respectively. Mean log frequency (SD) for Japanese words was 3.18 (0.62) per million for List 1 and 3.33 (0.58) per million for List 2, according to the BCCWJ database provided by the National Language Institute (https://pj.ninjal.ac.jp/corpus_center/en/). Auditory word stimuli were created by recording a female native Japanese speaker who read aloud each of the 40 FL words and their Japanese translations.

The behavioral paradigm consisted of several study–test cycles for FL word learning with two different study conditions (“writing” and “no-writing”). For each condition, participants first learned a list of 20 word-pairs in a study period and then recalled the meanings of FL words in a test period. In the writing condition, participants studied each FL word while spelling it out with the right index finger on a plastic board (Since the syllabic kana script is a highly transparent writing system having almost one-to-one correspondence between characters and syllables, participants could transcribe heard words into written forms exactly in the way they perceived the spoken stimuli.). In the no-writing condition, participants studied the target word while keeping the right hand relaxed on the board. The two conditions were performed in two separate sessions spaced at least 3 weeks from each other. Half of the participants started with the writing condition, while the other half with the no-writing condition. The effects of the lists (List 1 and List 2) and conditions (writing and no-writing) were counterbalanced across participants. All participants were blindfolded throughout the experiment.

A schematic diagram of the experimental procedure is illustrated in Fig. 1A. During the study period, participants heard each FL word and its Japanese translation presented with a stimulus-onset asynchrony of 3.5 s via circumaural headphones. On each trial, the same word pair was presented three times every 3.5 s. Trials were self-paced and separated by a brief chime sound and a silent interval of 3.5 s. During the test period immediately after the study period, participants were presented with spoken FL words and asked to translate them in Japanese. Spoken responses were recorded and analyzed offline. Participants received no feedback about response accuracy during the test period. This cycle of study and test was repeated outside the MRI scanner (see below) until participants achieved >50% accuracy in the test period (in fact, all but 2 participants exceeded 70% in accuracy after 2 cycles). To reach this criterion, blind participants performed 1.67 (0.98) cycles and 1.33 (0.49) cycles for the writing and no-writing conditions, respectively, whereas sighted participants needed 1.50 (0.79) cycles and 1.39 (0.49) cycles for the writing and no-writing conditions, respectively. Consequently, the number of study–test cycles prior to fMRI scanning differed neither between the two groups (*p* > 0.5) nor between the two conditions (*p* > 0.16).

Participants then lay in the MRI scanner and received one additional cycle of study and test delivered via closed-air circumaural earphones. We used only the last study–test cycle as the activation paradigm for fMRI to minimize anxiety stress and physical discomfort associated with MRI scanning. Spoken responses were recorded using an MRI-compatible microphone during the test period and analyzed as “immediate recall.” Handwriting movements were visually monitored via an in-bore camera system. Other experimental settings were all identical to those used for the preceding cycles outside the scanner. One week after fMRI scanning, participants further received the same recall test as “delayed recall” for each condition.

Since the accuracy data for the immediate and delayed recall tests did not meet the assumptions of normality (*p* = 1.79 × 10^–8^, Shapiro–Wilk test) and homogeneity of variance (*p* = 8.45 × 10^–5^, Levene test), these values were inverse-transformed to reduce variances and submitted to non-parametric aligned rank transformation ANOVA^32,33^ implemented in the ARTool package for R (https://cran.r-project.org).

### fMRI procedures

Imaging data were acquired using a Siemens Skyra 3 Tesla head scanner with a standard head coil optimized for a gradient echo–echo planar imaging (35 contiguous axial slices, thickness = 3 mm with 1 mm gap, repetition time = 3500 ms, echo time = 30 ms, acquisition time = 2500 ms, flip angle = 90°, field of view = 192 × 192 mm^2^). Note that the repetition time included a silent period of 1000 ms in which auditory word stimuli were delivered to participants. As described above, the writing and no-writing conditions were performed in two separate sessions spaced at least 3 weeks from each other. For each condition, participants received two runs, each of which included a study period and a test period. Head motion was minimized with tight foam cushions and elastic straps. Each run on average (SD) lasted 631 s (145) for the study and 283 s (46) for the test (see Supplementary Table 1 for further analyses). High-resolution anatomical images were obtained after the test period (224 sagittal slices, thickness = 1 mm without gap, repetition time = 2300 ms, echo time = 2.98 ms, inversion time = 900 ms, flip angle = 9°, field-of-view = 256 × 256 mm^2^).

### Statistics and reproducibility

For GLM analyses, functional imaging data were preprocessed and assessed using SPM12 (http://www.fil.ion.ucl.ac.uk/spm/). Images from each participant were corrected for head movements, normalized to the MNI template with a 2 × 2 × 2 mm^3^ voxel size and spatially smoothed with an isotropic Gaussian filter (5 mm width at half maximum). The extent of head movement differed neither between conditions nor between groups (Supplementary Table 2). These images were then high-pass filtered at 120 s and smoothed with a 4-s Gaussian kernel. For each period (study and test), a weighted-mean image for each condition (writing and no-writing) was computed by fitting each voxel time series with the known time series of trials convolved with a canonical hemodynamic response function and its temporal derivative for each participant. Six estimated parameters for head motion were included as regressors of no interest. The resulting contrast images were submitted to the second-level flexible factorial ANOVA that included effects of period (study vs. test), group (blind vs. sighted), and learning condition (writing vs. no-writing). Unless stated otherwise, statistical significance was assessed with voxel-level *p* < 0.001 and cluster-level *p* < 0.05 corrected for multiple comparisons with family-wise error.

We then performed ROI analyses to probe local neural response more closely for brain regions involved in word learning and handwriting. We first looked at the hippocampus that is well known to play a key role in adult vocabulary learning^51,52,65^. For each hemisphere, we constructed three ROIs by dividing the hippocampus into anterior, middle, and posterior segments according to the analysis procedure used in some recent fMRI studies^66,67^. The hippocampal voxels were extracted from the Harvard–Oxford subcortical atlas^31^ and divided into thirds along the anterior-to-posterior axis. Because of the known role of Exner’s area in handwriting, we created a 5-mm radius spherical ROI centered at the previously reported coordinates in the left PMd (−24, −4, 52)^23^. We also used a 5-mm radius ROI for the left occipitotemporal VWFA associated with orthographic codes of written words (−46, −56, −12)^35,68^, since this region may also play a role in novel word learning in adults^69,70^. For each ROI, neural effects of writing and group were assessed with voxel-level *p* < 0.05, corrected for multiple comparisons with family-wise error across the search volume.

We next performed PPI analyses^71^ to assess functional connectivity with Exner’s area across the study and test periods. In brief, PPI computes functional coupling between a seed ROI and all other regions induced by psychological context. To examine possible differences in the impact of writing on word learning between the blind and sighted people, we selected this group-by-condition interaction as a critical contrast for assessing functional connectivity during word learning. Regional responses per session per participant were extracted by calculating the principal eigenvariate across all voxels within a radius of 5-mm around the local maximum in Exner’s area identified in the whole-brain analysis (−24, 0, 48, see “Results”). For each participant, the PPI regressor was calculated as an element-by-element product of the neural response (physiological regressor) and a vector coding for the period × condition interaction (psychological regressor), i.e., the most critical contrast identified in the GLM analyses (see “Results”). A whole-brain GLM was computed using the three types of regressors for each participant. Contrast images representing the PPI were created for each participant and submitted to 2 × 2 ANOVA treating the effect of learning condition as a within-participant factor and the effect of group as a between-participant factor. We searched for brain regions showing group × condition interaction in functional connection strength with Exner’s area during the test period. A more lenient threshold (voxel *p* < 0.001, cluster size >10 voxels) was used to identify voxels showing the highest trend of interaction in whole-brain analysis. For each of the ROIs described above, the same effects were further assessed with voxel-level *p* < 0.05, corrected for multiple comparisons with family-wise error.

### Representational similarity analyses

RSAs were performed using PyMVPA (http://www.pymvpa.org/). We constructed three theoretical RDMs (motor, syllabic, and semantic; Fig. 1B) and calculated their correlations with neural activation patterns computed from fMRI signals. The motor RDM was constructed to characterize motor-level dissimilarity in writing movements between FL words. For each word, we first calculated the total number of strokes required for spelling out the whole string of characters. Motor dissimilarity between a pair of words was then calculated as the difference in stroke numbers divided by their sum. Pairwise comparisons of all possible word pairs yielded the motor RDM as a 40 × 40 symmetrical matrix of which the off-diagonal values represent the dissimilarity for each pair of words at the motor level. For the syllabic RDM, we likewise determined the total number of syllables for each FL word. Syllabic dissimilarity between a pair of words was defined as one minus the proportion of shared syllables between the two words against the total number of syllables summed across the two words, yielding a 40 × 40 matrix whose off-diagonal values represent pairwise dissimilarities between words at the syllable level. We constructed the semantic RDM by calculating semantic distance between words using the word similarity module of Sematch (https://gsi-upm.github.io/sematch), which allows measuring between-word semantic similarity according to the taxonomy provided by the WordNet (https://wordnet.princeton.edu/). Pairwise simple Mantel tests confirmed that the three theoretical RDMs did not correlate with each other (*p* > 0.1 for all).

To create neural RDMs, we constructed a GLM that included 40 regressors for FL words and 6 parameters for head motion for each period (study and test). For each period, we computed parameter estimates for each FL word using unsmoothed normalized images and created a single beta image per word per participant. These beta images served as whole-brain activation maps each corresponding to each FL word during each period. For each voxel, we constructed neural RDMs by calculating one minus Spearman rank correlations of all word pairs for each condition for each period for each participant. For each theoretical RDM, Spearman correlation with the neural RDM was then computed per condition per period per participant. The resulting correlation coefficients from all participants were converted to *Z*-values using Fisher transformation and submitted to 2 × 2 ANOVA treating the effect of learning condition as a within-participant factor and the effect of group as a between-participant factor. For each period, we first assessed the representational content of Exner’s area and left hippocampus identified in the GLM analyses described above. For each region, we used a more lenient statistical threshold (*p* < 0.005, uncorrected) than these GLM analyses to include more voxels in RSA (see “Results”).

Since our main interest was to explore neural correlates of the behavioral group × condition interaction during immediate recall, we performed two additional in-depth analyses for the test period. First, we selected six left hemisphere regions involved in phonological and semantic word processing and compared their representational content between the blind and sighted groups. Specifically, we constructed a 10-mm radius spherical ROI in the left MFG (−9, 48, 39), IFG (−51 24 12), anterior temporal lobe (−39, −6, 39), anterior superior temporal gyrus (−51, 9, −12), pMTG (−56, −36, −2), and SMG (−48, −64, 34) according to the ROI selection procedure used in a recent fMRI study, which probed the left hemisphere language network^72^. It is important to note that all these structures are shown to participate in novel word learning^51,52^. For each ROI, the *Z* converted correlation coefficients per condition were collapsed for each participant and submitted to one-sample *t* test to determine whether overall correlation strength with neural RDMs differed from zero across participants. We then assessed the same correlation coefficients using 2 × 2 ANOVA treating the effect of learning condition as a within-participant factor and the effect of group as a between-participant factor. All *p* values were corrected for multiple comparisons for each ROI for each period using false-discovery rate. Second, we used whole-brain searchlight RSA^73^ to search for other neural systems showing sensitivity to the three theoretical RDMs. A spherical searchlight with a radius of two voxels was moved within the voxels with a gray matter probability >0.33 for each condition for each participant. The resulting *r* maps per condition per participant were converted to *Z* maps using Fisher transformation and submitted to the ANOVA model in SPM12, which included the effects of condition and group as described above.

### Reporting summary

Further information on research design is available in the Nature Research Reporting Summary linked to this article.

## Supplementary information

Supplementary Information

Description of Additional Supplementary Files

Supplementary Data 1

Reporting Summary

## Data Availability

Behavioral and MRI data analyzed in the present study are archived in the Section of Systems Neuroscience, NRCD Research Institute and available from the corresponding author on reasonable request. All source data underlying the plots shown in figures are provided in Supplementary Data 1.

## Code availability

All codes used for data analysis are available from the corresponding author on reasonable request.
