# Supplementary material for: Graphomotor memory in Exner’s area enhances word learning in the blind
Source: Commun Biol. 2021 Apr 6;4:443. doi: 10.1038/s42003-021-01971-z (PMC8024258; doi:10.1038/s42003-021-01971-z)
Supplement: Supplementary file 1 — Supplementary Information [file 42003_2021_1971_MOESM1_ESM.pdf]

Supplementary Table 1. Duration (s) of fMRI runs during the study and test periods.

	Blind		Sighted	
	Writing	No-writing	Writing	No-writing
Study	624 (86)	623 (96)	669 (209)	604 (129)
Test	276 (39)	263 (32)	286 (31)	299 (65)

Mean duration (SD) for each period is presented per condition per group. For the study period, we assessed the effects of condition and group on run durations using 2 x 2 ANOVA and confirmed that these effects and their interaction were all non-significant (all p s > 0.1). As for the test period, the effects of group and condition and their interaction were also non-significant (all p s > 0.1). Accordingly, the overall duration of fMRI runs differed neither between groups nor between conditions.

Supplementary Table 2. Head motion during fMRI scanning.

			Blind		Sighted	
			Writing	No-writing	Writing	No-writing
Study	Translation (mm)	*x*	0.31 (0.12)	0.30 (0.12)	0.54 (0.48)	0.37 (0.30)
		y	0.32 (0.26)	0.31 (0.24)	0.52 (0.37)	0.55 (0.77)
		z	0.65 (0.29)	0.72 (0.42)	1.04 (0.73)	1.00 (1.17)
	Rotation (degrees)	*pitch*	0.01 (0.00)	0.02 (0.01)	0.01 (0.01)	0.01 (0.01)
		yaw	0.00 (0.00)	0.01 (0.01)	0.01 (0.00)	0.01 (0.00)
		roll	0.01 (0.00)	0.01 (0.01)	0.01 (0.00)	0.01 (0.01)
Test	Translation (mm)	*x*	0.22 (0.11)	0.18 (0.10)	0.27 (0.20)	0.34 (0.31)
		y	0.28 (0.17)	0.26 (0.17)	0.41 (0.22)	0.38 (0.42)
		z	0.54 (0.37)	0.48 (0.17)	0.75 (0.43)	0.71 (0.54)
	Rotation (degrees)	*pitch*	0.01 (0.01)	0.02 (0.01)	0.01 (0.01)	0.01 (0.01)
		yaw	0.00 (0.00)	0.00 (0.00)	0.01 (0.00)	0.01 (0.00)
		roll	0.00 (0.01)	0.01 (0.00)	0.00 (0.00)	0.01 (0.01)

For each of the six displacement parameters (x, y, z, pitch, yaw, roll), the maximum for each run was averaged (SD) across participants per condition per period per group. For each parameter, the effects of condition and group on head motion were assessed using 2 x 2 ANOVA (p values corrected with FDR for each period). For the study period, we confirmed that the two main effects and their interaction were non-significant for each of the six parameters ($p > 0.5$ for all). As for the test period, the effects of group and condition and their interaction were also non-significant for all parameters ($p > 0.\ 5$ for all).
