# Supplementary material for: Graphomotor memory in Exner’s area enhances word learning in the blind
Source: Commun Biol. 2021 Apr 6;4:443. doi: 10.1038/s42003-021-01971-z (PMC8024258; doi:10.1038/s42003-021-01971-z)
Supplement: Supplementary file 2 — Description of Additional Supplementary Files [file 42003_2021_1971_MOESM2_ESM.pdf]

Description of Additional Supplementary Files

File name: Supplementary Data 1

Description: Source data underlying the plots shown in figures.
