# Supplementary material for: Graphomotor memory in Exner’s area enhances word learning in the blind
Source: Commun Biol. 2021 Apr 6;4:443. doi: 10.1038/s42003-021-01971-z (PMC8024258; doi:10.1038/s42003-021-01971-z)
Supplement: Supplementary file 4 — Reporting Summary [file 42003_2021_1971_MOESM4_ESM.pdf]

nature research

Corresponding author(s): Kimihiro Nakamura

Last updated by author(s): Mar 30, 2021

Reporting Summary

Nature Research wishes to improve the reproducibility of the work that we publish. This form provides structure for consistency and transparency in reporting. For further information on Nature Research policies, see our Editorial Policies and the Editorial Policy Checklist.

Statistics

For all statistical analyses, confirm that the following items are present in the figure legend, table legend, main text, or Methods section.

n/a	Confirmed	
☐	☒	The exact sample size (*n*) for each experimental group/condition, given as a discrete number and unit of measurement
☐	☒	A statement on whether measurements were taken from distinct samples or whether the same sample was measured repeatedly
☐	☒	The statistical test(s) used AND whether they are one- or two-sided<br>*Only common tests should be described solely by name; describe more complex techniques in the Methods section.*
☐	☒	A description of all covariates tested
☐	☒	A description of any assumptions or corrections, such as tests of normality and adjustment for multiple comparisons
☐	☒	A full description of the statistical parameters including central tendency (e.g. means) or other basic estimates (e.g. regression coefficient) AND variation (e.g. standard deviation) or associated estimates of uncertainty (e.g. confidence intervals)
☐	☒	For null hypothesis testing, the test statistic (e.g. *F*, *t*, *r*) with confidence intervals, effect sizes, degrees of freedom and *P* value noted<br>*Give P values as exact values whenever suitable.*
☒	☐	For Bayesian analysis, information on the choice of priors and Markov chain Monte Carlo settings
☒	☐	For hierarchical and complex designs, identification of the appropriate level for tests and full reporting of outcomes
☐	☒	Estimates of effect sizes (e.g. Cohen's *d*, Pearson's *r*), indicating how they were calculated

Our web collection on statistics for biologists contains articles on many of the points above.

Software and code

Policy information about availability of computer code

Data collection	Presentation, Neurobehavioral Systems (https://www.neurobs.com/)
Data analysis	R (https://www.r-project.org/)<br>SPM12 (https://www.fil.ion.ucl.ac.uk/spm/software/spm12/)<br>PyMVPA (http://www.pymvpa.org/)

For manuscripts utilizing custom algorithms or software that are central to the research but not yet described in published literature, software must be made available to editors and reviewers. We strongly encourage code deposition in a community repository (e.g. GitHub). See the Nature Research guidelines for submitting code & software for further information.

Data

Policy information about availability of data

All manuscripts must include a data availability statement. This statement should provide the following information, where applicable:
- Accession codes, unique identifiers, or web links for publicly available datasets
- A list of figures that have associated raw data
- A description of any restrictions on data availability

The behavioral and MRI datasets analyzed in the present study are available from the corresponding author on reasonable request.

Field-specific reporting

Please select the one below that is the best fit for your research. If you are not sure, read the appropriate sections before making your selection.

☒ Life sciences ☐ Behavioural & social sciences ☐ Ecological, evolutionary & environmental sciences

For a reference copy of the document with all sections, see nature.com/documents/nr-reporting-summary-flat.pdf

Life sciences study design

All studies must disclose on these points even when the disclosure is negative.

Sample size	Twelve late-blind participants were recruited at the Training Center for Independent Living, National Rehabilitation Center for Persons with Disabilities. A separate age- and education-matched group of 18 sighted participants were also recruited. A total of 30 participants in the present study exceed the typical sample size of fMRI research (e.g., Friston et al, Neuroimage 1999)
Data exclusions	No data were excluded from analyses.
Replication	The reproducibility of the experimental findings was verified by running data analysis multiple times; all of these attempts produced consistent results.
Randomization	Random allocation of participants to experimental groups is not relevant to the present study, because our effects of interest are modeled as within-participant factors (i.e., each participant received all conditions, of which the order was counterbalanced across participants).
Blinding	Blinding was not possible because both investigators and participants must understand the nature of the behavioral task used for fMRI.

Reporting for specific materials, systems and methods

We require information from authors about some types of materials, experimental systems and methods used in many studies. Here, indicate whether each material, system or method listed is relevant to your study. If you are not sure if a list item applies to your research, read the appropriate section before selecting a response.

Materials & experimental systems

n/a	Involved in the study	
☒	☐	Antibodies
☒	☐	Eukaryotic cell lines
☒	☐	Palaeontology and archaeology
☒	☐	Animals and other organisms
☐	☒	Human research participants
☒	☐	Clinical data
☒	☐	Dual use research of concern

Methods

n/a	Involved in the study	
☒	☐	ChIP-seq
☒	☐	Flow cytometry
☐	☒	MRI-based neuroimaging

Human research participants

Policy information about studies involving human research participants

Population characteristics	see above.
Recruitment	Twelve late-blind participants were recruited at the Training Center for Independent Living, National Rehabilitation Center for Persons with Disabilities. A separate age- and education-matched group of 18 sighted participants were also recruited.
Ethics oversight	The protocol of this study was approved by the institutional ethics committee of the National Rehabilitation Center for Persons with Disabilities.

Note that full information on the approval of the study protocol must also be provided in the manuscript.

Magnetic resonance imaging

Experimental design

Design type	task; event-related
Design specifications	During the study period, participants heard each of 40 foreign-language word and their Japanese translation presented with a stimulus-onset-asynchrony of 3.5 s. On each trial, the same word pair was presented three times every 3.5 s. Trials were self-paced and separated by a brief chime sound and a silent interval of 3.5 s. During the test period

	immediately after the study period, participants were presented with spoken foreign-language words and asked to translate them in Japanese.
Behavioral performance measures	Spoken responses during immediate recall were recorded for each condition for each participant. Participants performed the task as expected, since blind participants identified word meanings with accuracy level (SD) of 92.50 (9.65) % in the writing condition and 92.08 (10.33) % in the no-writing condition, respectively. Sighted participants performed the same task with accuracy level of 90.27 (11.04) % in the writing condition and 95.28 (6.06) % in the no-writing condition, respectively.

Acquisition

Imaging type(s)	functional
Field strength	3 Tesla
Sequence & imaging parameters	35 contiguous axial slices, thickness = 3 mm with 1 mm gap, repetition time = 3500 ms, echo time = 30 ms, acquisition time = 2500 ms, flip angle = 90º, field-of-view =192 x 192 mm2
Area of acquisition	whole-brain scan

Diffusion MRI ☐ Used ☒ Not used

Preprocessing

Preprocessing software	SPM12 (http://www.fil.ion.ucl.ac.uk/spm/)
Normalization	Images from each participant were corrected for head movements, normalized to the Montreal Neurological Institute template with a 2 x 2 x2 mm3 voxel size using the linear and non-linear transformation procedure implemented in SPM12.
Normalization template	MNI305
Noise and artifact removal	Images from each participant were corrected for head movements, normalized to the Montreal Neurological Institute template with a 2 x 2 x2 mm3 voxel size and spatially smoothed with an isotropic Gaussian filter (5mm width at half maximum). These images were then high-pass filtered at 120 s and smoothed with a 4 s Gaussian kernel.
Volume censoring	SPM12 (http://www.fil.ion.ucl.ac.uk/spm/)

Statistical modeling & inference

Model type and settings	In the first-level univariate analysis, we computed a weighted-mean image for each condition (writing and no-writing) for each period (study and test) by fitting each voxel time-series with the known time-series of trials convolved with a canonical hemodynamic response function and its temporal derivative for each participant. In the second-level analysis, we assessed the effects of period (study vs. test), group (blind vs. sighted) and learning condition (writing vs. no-writing) using flexible factorial ANOVA implemented in SPM12, which treated these effects as fixe-effects and participants as random effects, respectively. In RSA, we constructed three theoretical representational dissimilarity matrices (motor, syllabic and semantic RDMs). For each RDM, Spearman correlation with the neural RDM for each ROI was then computed per condition per period per participant.
Effect(s) tested	In both univariate analyses and RSAs, we examined neural correlates of the interaction between learning condition (writing vs. no-writing) and group (blind vs. sighted).

Specify type of analysis: ☐ Whole brain ☐ ROI-based ☒ Both

Anatomical location(s)	We constructed a 10-mm radius spherical ROI in the left middle frontal gyrus (MFG; −9, 48, 39), inferior frontal gyrus (IFG; -51 24 12) anterior temporal lobe (ATL; -39, -6, 39), anterior superior temporal gyrus (aSTG; -51, 9, -12), posterior middle temporal gyrus (pMTG; -56, -36, -2) and supramarginal gyrus (SMG; -48, -64, 34) according to the ROI selection procedure used in a recent fMRI study which probed the left-hemisphere language network (Jackson et al, J Neurosci 2016).
Statistic type for inference (See Eklund et al. 2016)	Unless stated otherwise, statistical significance was assessed with voxel-level p < 0.001 and cluster-level p < 0.05 corrected for multiple comparisons with family-wise error.
Correction	FWE

Models & analysis

n/a	Involved in the study
☐	☒ Functional and/or effective connectivity
☒	☐ Graph analysis
☐	☒ Multivariate modeling or predictive analysis

Functional and/or effective connectivity	We performed psychophysiological interaction (PPI) analyses to assess functional connectivity with Exner's

area across the study and test periods. For each participant, the PPI regressor was calculated as an element-by-element product of the neural response (physiological regressor) and a vector coding for the period x condition interaction (psychological regressor), i.e., the most critical contrast identified in the GLM analyses. A whole-brain general linear model was computed using the three types of regressors for each participant..

Multivariate modeling and predictive analysis

We performed representational similarity analysis to assess the representational content of the brain regions identified in GLM analyses, i.e., Exner's area and the left hippocampus. Three theoretical representational dissimilarity matrices (RDMs) were created to characterize the motor-, syllabic- and semantic-level dissimilarity between foreign language (FL) words. We also computed parameter estimates for each FL word, which served as whole-brain activation maps for each word. Neural RDMs were then created by calculating one minus Spearman rank correlations of all word pairs for each condition for each participant. For each theoretical RDM, Spearman correlation with the neural RDM was computed per condition per participant.
